# Proprioceptive deficits and postural instability in adolescent idiopathic scoliosis: a comparative study of balance control and key predictors

**DOI:** 10.3389/fped.2025.1595125

**Published:** 2025-06-12

**Authors:** Ghada Koura, Ahmed Mohamed F. Elshiwi, Ravi Shankar Reddy, Saud M. Alrawaili, Zeinab A. Ali, Mazen Abdullah N. Alshahrani, Amer Abdullah M. Alshahri, Sultan Sarhan Z. Al-Ammari

**Affiliations:** ^1^ Department of Medical Rehabilitation Sciences, College of Applied Medical Sciences, King Khalid University, Abha, Saudi Arabia; ^2^ Faculty of Physical Therapy, Cairo University, Cairo, Egypt; ^3^ Department of Physical Therapy, Saudi German Hospital, Khamis Mushait, Saudi Arabia; ^4^ Department of Health and Rehabilitation Sciences, Prince Sattam Bin Abdulaziz University, Al-Kharj, Saudi Arabia; ^5^ Department of Physical Therapy and Health Rehabilitation, College of Applied Medical Science, Jouf University, Al-Jouf, Saudi Arabia

**Keywords:** adolescent idiopathic scoliosis, proprioception, postural stability, balance control, center of pressure, rehabilitation

## Abstract

**Background:**

Adolescent idiopathic scoliosis (AIS) is associated with postural instability, which may be influenced by proprioceptive deficits. While previous studies have examined balance impairments in scoliosis, the extent to which proprioception errors contribute to postural instability remains unclear. Understanding this relationship is crucial for developing targeted rehabilitation strategies.

**Objectives:**

This study aimed to assess proprioceptive accuracy and postural stability in individuals with AIS compared to healthy controls and identify key predictors of postural instability.

**Methods:**

This cross-sectional study included 60 participants (30 with AIS, 30 controls). Postural stability was assessed using the Biodex Balance System (BBS), measuring Overall Stability Index (OSI), Anterior-Posterior Stability Index (APSI), Medial-Lateral Stability Index (MLSI), Center of Pressure (COP) displacement, and Reaction Time. Lumbar proprioception errors in flexion and extension were evaluated using an inclinometer-based joint repositioning test.

**Results:**

The AIS group showed significantly greater COP displacement (*p* = 0.013) and lumbar proprioception errors in flexion (*p* = 0.021) and extension (*p* = 0.004) compared to controls. Regression analysis identified proprioception errors and COP displacement as significant predictors of postural instability (R² = 0.647).

**Conclusion:**

Individuals with scoliosis exhibit significant proprioceptive deficits, which strongly correlate with postural instability. These findings highlight the importance of proprioceptive training in scoliosis rehabilitation to improve balance control.

## Introduction

Postural stability is a complex function that relies on the integration of sensory inputs from the visual, vestibular, and proprioceptive systems to maintain balance and coordinate movement (1). Proprioception, which refers to the body's ability to sense joint position and movement, plays a fundamental role in postural control by providing continuous feedback to the central nervous system for balance adjustments (2). Disruptions in proprioceptive feedback can lead to impaired motor coordination, increased postural sway, and balance instability (2). While postural control mechanisms are typically well-regulated in healthy individuals, musculoskeletal conditions such as adolescent idiopathic scoliosis (AIS) can disrupt sensorimotor integration, potentially leading to balance impairments (2, 3). Given that scoliosis is characterized by spinal deformity and musculoskeletal asymmetry, it may alter proprioceptive feedback mechanisms and compromise postural control (3). Emerging evidence suggests that systemic factors such as nutritional deficiencies may also contribute to postural instability and scoliosis development. Specifically, vitamin D deficiency has been linked to impaired musculoskeletal function and reduced postural balance, potentially increasing susceptibility to spinal deformities in adolescents (4). Scaturro et al. (4) reported that lower serum vitamin D levels were associated with postural instability and scoliosis severity, underscoring the importance of considering metabolic and nutritional aspects in the etiopathogenesis of AIS and related balance impairments (4).

Adolescent idiopathic scoliosis is a three-dimensional spinal deformity that affects postural alignment and musculoskeletal function (5). In addition to structural changes, individuals with AIS often exhibit neuromuscular adaptations that may influence balance control (6). Previous research has suggested that scoliosis may impact postural stability due to asymmetrical muscle activation and altered biomechanical loading on the spine and lower limbs (6). These changes can modify the way sensory information is processed, leading to deficits in proprioception and balance regulation (7). Studies have reported increased postural sway and instability in individuals with scoliosis (8), particularly when performing dynamic tasks or maintaining balance in challenging conditions. However, despite the well-documented postural impairments in scoliosis, the extent to which proprioceptive deficits contribute to postural instability remains unclear (8). Since proprioception is critical for maintaining stability, investigating its role in scoliosis-related balance impairments is essential for understanding the underlying mechanisms of postural control dysfunction in this population (9). Neurophysiologically, AIS is associated with impaired sensorimotor integration, potentially due to disrupted afferent signaling from paraspinal mechanoreceptors (8). Structural asymmetry in the spine may alter load distribution and affect proprioceptive input, leading to maladaptive central processing of joint position sense (8). These neurobiological mechanisms underpin the observed deficits and justify the need for focused proprioceptive assessment (8).

Although postural instability has been observed in individuals with scoliosis, research on the relationship between proprioception and balance impairments remains limited (9). Some studies have suggested that scoliosis patients rely more on visual and vestibular input to compensate for proprioceptive deficits, but the degree to which proprioception errors contribute to postural instability has not been thoroughly investigated (8, 10). Additionally, most studies have focused on global postural stability measures without specifically examining how proprioceptive errors in the lumbar region influence balance control (11). Given that the lumbar spine plays a crucial role in postural adjustments, a deeper understanding of lumbar proprioceptive deficits in scoliosis is needed (11). A recent systematic review and meta-analysis by Lau et al. (12) emphasized the prevalence of proprioceptive deficits in AIS but noted inconsistencies in measurement protocols. Similarly, Sluga et al. (13) reviewed various exercise-based interventions and highlighted the emerging role of proprioceptive training in improving postural balance, though evidence remains inconclusive due to heterogeneity in methodologies. Furthermore, previous studies have not consistently identified the key predictors of postural instability in scoliosis, making it difficult to determine whether proprioceptive deficits or other factors, such as reaction time and postural sway, play a more significant role in balance impairments (12, 14). Addressing this research gap is essential for developing targeted interventions to improve postural stability in individuals with scoliosis.

This study aimed to (1) compare lumbar proprioception accuracy between individuals with AIS and healthy controls, (2) evaluate differences in postural stability parameters, and (3) identify key predictors of postural instability, with a focus on proprioceptive errors. The study hypothesized that individuals with AIS would exhibit greater lumbar proprioception errors and increased postural instability compared to healthy controls, and that these proprioceptive deficits would significantly predict balance impairments.

## Methods

### Study design

This cross-sectional study was conducted in accordance with the CONSORT guidelines. Ethical approval was obtained from the King Khalid University Ethical Committee (ECM#2023-2105). The study took place at the Balance Laboratory of the Faculty of Physical Therapy, King Khalid University, between June 2023 and February 2024. Prior to data collection, all participants and their parents were fully informed about the study's objectives, procedures, and their right to withdraw at any time. Written informed consent was obtained from all participants and their legal guardians before enrollment in the study.

### Sample size calculation

The sample size for this study was determined using G*Power 3.1 statistical software to ensure adequate power for detecting significant differences in postural stability and proprioception measures between individuals with scoliosis and healthy controls. The calculation was based on previously published studies that investigated proprioception errors and postural stability deficits in scoliosis (14). A two-tailed independent *t*-test was chosen as the statistical test, with an effect size (Cohen's d) of 0.80, representing a large effect based on prior research findings. The alpha level (α) was set at 0.05, and the power (1-β) was set at 0.80, ensuring an 80% probability of detecting true differences between groups. Using these parameters, the estimated minimum required sample size was 26 participants per group. To account for potential dropouts and variability in measurements, the final sample size was increased to 30 participants per group, resulting in a total of 60 participants.

### Participants

Participants in this study were individuals diagnosed with AIS and age-matched healthy controls. The scoliosis group was recruited from orthopedic and rehabilitation clinics, while the control group consisted of volunteers from the general population who met the study's eligibility criteria. Diagnosis of AIS was confirmed by an orthopedic specialist based on radiographic evidence of a spinal curvature with a Cobb angle of at least 10 degrees, without any known underlying neuromuscular or congenital causes (15). The selection process ensured that all participants were within the adolescent age range and had no prior history of spinal surgery or medical conditions that could influence balance or proprioception. The Inclusion and the exclusion criteria are followed as mentioned in Table 1.

**Table 1:** Inclusion and exclusion criteria.

Inclusion criteria	Exclusion criteria
Aged 14–21 years	History of spinal surgery
Diagnosed with AIS (Cobb angle 10°–40°)	Leg length discrepancy >2 cm
No neurological/musculoskeletal disorders (except scoliosis)	Vestibular dysfunction
Able to stand and perform balance tests without assistance	Neuromuscular or orthopedic conditions affecting posture
Informed consent from participants and guardians	Participation in proprioception/balance training in past 6 months
Normal or corrected-to-normal vision	Pregnancy or cognitive impairment

Control group met the same criteria except scoliosis diagnosis and related structural deformities.

**Figure 1:**
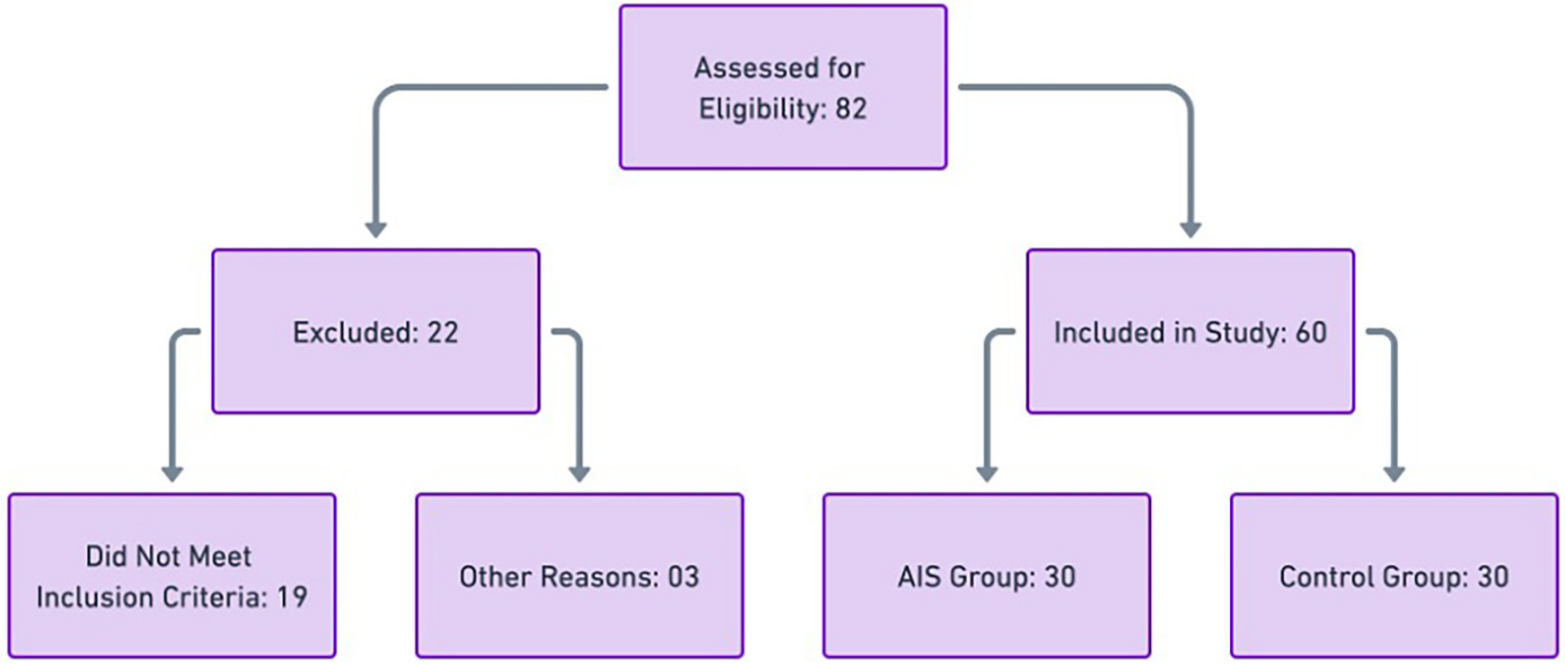
Participant flow diagram illustrating participant recruitment, screening, exclusion, and final group allocation.

Participant recruitment was conducted through referrals from healthcare providers, university health centers, and community advertisements. Individuals meeting the eligibility criteria were invited for an initial screening, which included a physical examination and radiographic confirmation for the scoliosis group. All eligible participants were provided with detailed information about the study, and written informed consent was obtained from both participants and their legal guardians before enrollment. The Participant Flow Diagram illustrating participant recruitment, screening, exclusion, and final group allocation is presented in Figure 1.

### Variables and methods of assessment

This study examined postural stability and proprioception in individuals with AIS compared to healthy controls. The primary outcome variables included postural stability indices (overall stability index, anterior-posterior stability index, and medial-lateral stability index), center of pressure displacement, reaction time, total balance test duration, and proprioception errors in lumbar flexion and extension. All measurements were conducted in a controlled laboratory setting using standardized assessment tools to ensure accuracy and reproducibility.

### Postural stability assessment

**Figure 2:**
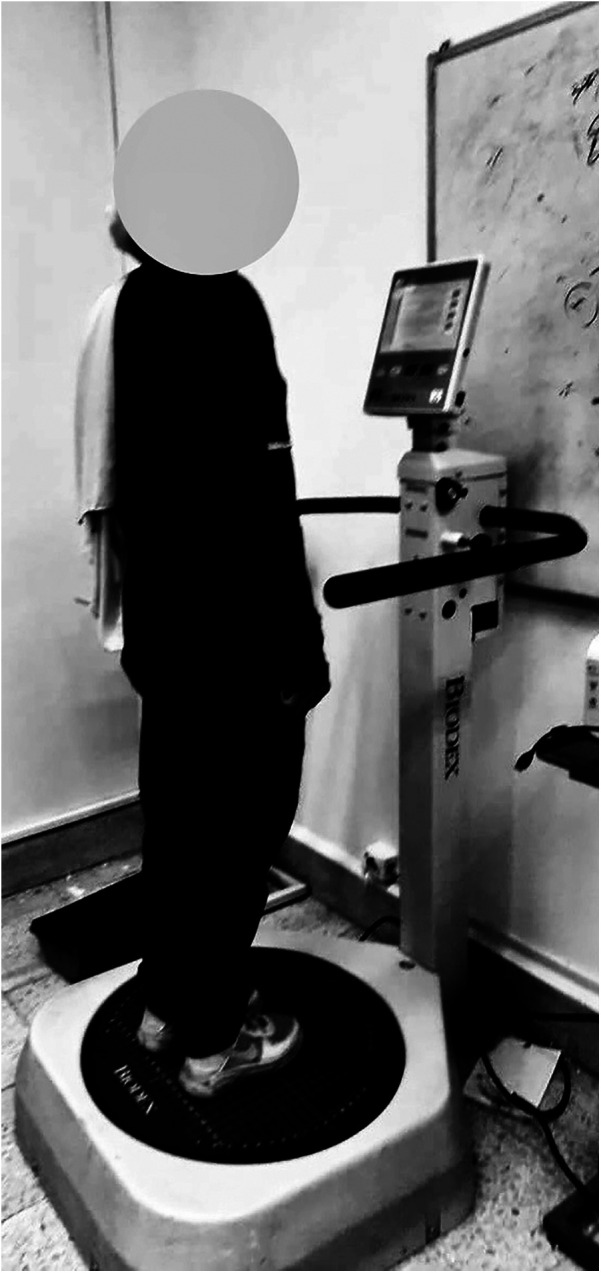
Postural stability was assessed using the biodex balance system.

Postural stability was assessed using the computerized is (Biodex Inc., Shirley, NY, USA), a validated multiaxial device designed to evaluate postural balance (Figure 2) (16). The BBS operates by adjusting the stability of a circular force platform, which allows multiplanar movement and can tilt up to 20° in any direction, simulating postural challenges. The stability level was set at level four, providing a moderate challenge while ensuring participant safety, in accordance with previous studies (17). Participants stood barefoot on the platform with their feet positioned according to the manufacturer's guidelines. They were instructed to cross their arms over their chest and focus on a visual feedback screen, where a cursor represented their center of mass. Their task was to maintain the cursor as close as possible to a central target while the platform moved. Before formal testing, each participant underwent a one-minute orientation session to familiarize themselves with the device. The postural stability test measured three key stability indices: the Overall Stability Index (OSI), representing total postural sway and overall balance control (18); the Anterior-Posterior Stability Index (APSI), which assesses balance control in the sagittal plane; and the Medial-Lateral Stability Index (MLSI), which evaluates balance control in the frontal plane. Higher values in these indices indicate greater postural instability and a higher fall risk (18). Each participant completed three trials, each lasting 20 s, with a 10 s rest period between trials. The BBS automatically calculated the mean score across trials for each stability index to ensure reliable assessment (18). In addition to postural stability indices, center of pressure (COP) displacement was recorded in millimeters (mm) using the BBS. This measure reflects the extent of postural sway, with greater values indicating poorer balance control (18). Reaction time (measured in milliseconds) was also recorded to evaluate the participants' ability to respond to balance perturbations. Both COP displacement and reaction time values were averaged across three trials for each participant. The Limits of Stability (LOS) test was used to assess voluntary postural control and movement efficiency. Participants were required to lean toward a target on the screen while standing on the unstable platform. Using their ankles as the primar*y* axis of rotation, they moved a cursor on the screen by shifting their center of pressure. The goal was to reach each of the eight targets, which were positioned 45 degrees apart, as quickly and accurately as possible while maintaining a straight body posture. The LOS test recorded total time (seconds), representing the duration required to reach all targets, and directional control (%), which measures movement accuracy, where 100% indicates a direct path from the center to the target. The test was performed three times per participant, and the mean value was used for analysis. The target placement was standardized at 50% of the LOS, ensuring consistency across participants. This test provided additional insight into dynamic postural control and voluntary balance adjustments.

### Lumbar proprioception assessment

Lumbar proprioception, defined as the ability to perceive and control the position and movement of the lumbar spine, was assessed using a standardized joint reposition error (JRE) protocol (19). The assessment was conducted in a quiet and controlled laboratory setting to minimize external distractions. Participants were blindfolded to eliminate visual input and ensure reliance on proprioceptive feedback during the test. Before data collection, each participant was familiarized with the procedure and performed a practice trial under the supervision of a trained examiner. The assessment focused on lumbar JRE in flexion and extension (20). Measurements were obtained using a dual-inclinometer method, a validated and reliable technique for assessing proprioceptive accuracy of the lumbar spine. The primary inclinometer was placed at the T12 level on the chest, while the secondary inclinometer was positioned at the S1 level on the pelvis to capture lumbar movement. Participants were instructed to stand in their self-selected neutral spine position, which was recorded as the starting reference (20). For each movement, the examiner guided the participant into the target position, which was set at 50% of their available range of motion (ROM) for lumbar flexion or extension. The participant maintained this position for five seconds to enhance kinesthetic awareness before returning to the starting position. They were then asked to actively reposition their lumbar spine to the memorized target position without assistance. Once they believed they had reached the target, they verbally indicated completion by saying “YES”. The JRE was recorded as the difference between the target angle and the participant's reproduced angle, measured in degrees. Higher values indicated greater proprioceptive error and reduced repositioning accuracy. Each participant completed three trials per movement, and the mean value of the three trials was used for statistical analysis. This method ensured an objective and reproducible assessment of lumbar proprioceptive function in both scoliosis and control groups.

All assessments were conducted by trained evaluators to minimize inter-rater variability. Participants were given sufficient practice trials before formal testing to ensure familiarity with the procedures. Data collection adhered to a strict protocol, with environmental conditions controlled to prevent external influences on postural control measurements. These standardized methods allowed for a reliable comparison between scoliosis and control groups, providing objective measures of postural stability and proprioception deficits in AIS. Although the use of a stabilometric platform could assess static postural stability, the Biodex Balance System was selected to enable a more dynamic evaluation of balance performance under postural perturbations, aligning with the study's focus on functional postural control.

### Statistical analyses

Statistical analyses were conducted using SPSS version 24, with a significance level set at *p* < 0.05. As the data followed a normal distribution, parametric tests were applied. Descriptive statistics were calculated for all demographic, clinical, postural stability, and proprioception variables, including means and standard deviations. Independent *t*-tests were performed to compare differences between the control and scoliosis groups. Pearson correlation analysis examined relationships between postural stability indices and proprioception errors. Multiple regression analysis was conducted to identify significant predictors of postural stability, assessing the contribution of proprioception errors, reaction time, postural sway, and center of pressure displacement. The confidence intervals were reported for all comparisons to ensure statistical precision and reliability. As this was an exploratory study focused on predefined primary variables, corrections for multiple comparisons were not applied. However, interpretation of *p*-values was conducted with caution to avoid Type I error inflation.

## Results

The demographic characteristics between the scoliosis and control groups were comparable, with no statistically significant differences observed in age, height, weight, BMI, or physical activity levels, indicating appropriate group matching for general physical parameters (Table 2). Clinical parameters specific to scoliosis—including Cobb angle, leg length discrepancy, pelvic tilt, spinal rotation, and duration of AIS—were assessed only in the scoliosis group and not applicable to the control group. These findings confirm that the groups were demographically homogeneous, while the scoliosis group presented the expected clinical features associated with spinal deformity.

**Table 2:** Demographic and clinical characteristics.

Variable	Control Mean ± SD (*n* = 30)	Scoliosis Mean ± SD (*n* = 30)	95% CI Lower	95% CI Upper	*p*-value
Age (years)	19.50 ± 2.30	19.40 ± 2.50	−1.12	1.32	0.872
Height (cm)	162.60 ± 5.40	161.80 ± 5.60	−1.98	3.58	0.575
Weight (kg)	60.50 ± 8.80	58.70 ± 9.10	−2.73	6.33	0.439
BMI (kg/m²)	22.00 ± 2.10	21.80 ± 2.20	−0.89	1.29	0.720
Cobb Angle (°)	N/A	28.50 ± 5.80	-	-	-
Leg Length Discrepancy (cm)	N/A	1.20 ± 0.50	-	-	-
Pelvic Tilt (°)	N/A	5.60 ± 1.20	-	-	-
Spinal Rotation (°)	N/A	7.80 ± 1.50	-	-	-
Duration of AIS (years)	N/A	4.20 ± 1.10	-	-	-
Physical Activity Level (METs)	8.50 ± 1.20	7.80 ± 1.50	0.01	1.39	0.051

AIS, adolescent idiopathic scoliosis; BMI, body mass index; BBS, biodex balance system; METs, metabolic equivalent of task; SD, standard deviation; CI, confidence interval; *p*-value, probability value; cm, centimeters; kg, kilograms;°, degrees; N/A, not assessed.

Compared to controls, individuals with scoliosis demonstrated significantly greater center of pressure displacement and increased lumbar proprioception errors in both flexion and extension, indicating impaired postural control and reduced proprioceptive accuracy (Table 3). No significant differences were observed between groups in overall, anterior-posterior, or medial-lateral stability indices, nor in total time, reaction time, or directional control measures, suggesting that gross balance parameters remained comparable. These findings highlight specific sensorimotor impairments rather than generalized balance dysfunction in the scoliosis group.

**Table 3:** Comparison of postural stability and proprioception measures between control and scoliosis groups.

Variable	Control Mean ± SD	Scoliosis Mean ± SD	*t*-value	*p*-value	95% CI Lower	95% CI Upper
Overall Stability Index	5.89 ± 1.36	6.39 ± 2.32	−1.02	0.313	−1.46	0.46
A/P Stability Index	4.59 ± 1.13	5.10 ± 1.75	−1.34	0.185	−1.26	0.24
M/L Stability Index	3.65 ± 1.32	4.26 ± 1.69	−1.56	0.125	−1.38	0.16
Total Time (sec)	197.20 ± 75.22	176.10 ± 73.82	1.10	0.277	−16.61	58.81
Reaction Time (ms)	300.40 ± 45.30	320.20 ± 50.60	−1.60	0.116	−44.10	4.50
Overall Directional Control (%)	8.70 ± 4.82	9.43 ± 5.82	−0.53	0.599	−3.43	1.97
Center of Pressure Displacement (mm)	5.40 ± 1.10	6.20 ± 1.30	−2.57	0.013	−1.41	−0.19
Lumbar Proprioception Error in Flexion (°)	2.50 ± 0.85	3.10 ± 1.10	−2.36	0.021	−1.10	−0.10
Lumbar Proprioception Error in Extension (°)	3.20 ± 0.90	4.00 ± 1.15	−3.00	0.004	−1.32	−0.28

A/P, anterior-posterior; M/L, medial-lateral; SD, standard deviation; CI, confidence interval; *p*-value, probability value; BBS, biodex balance system; sec, seconds; mm, millimeters; ms, milliseconds; METs, metabolic equivalent of task;°, degrees.

**Figure 3:**
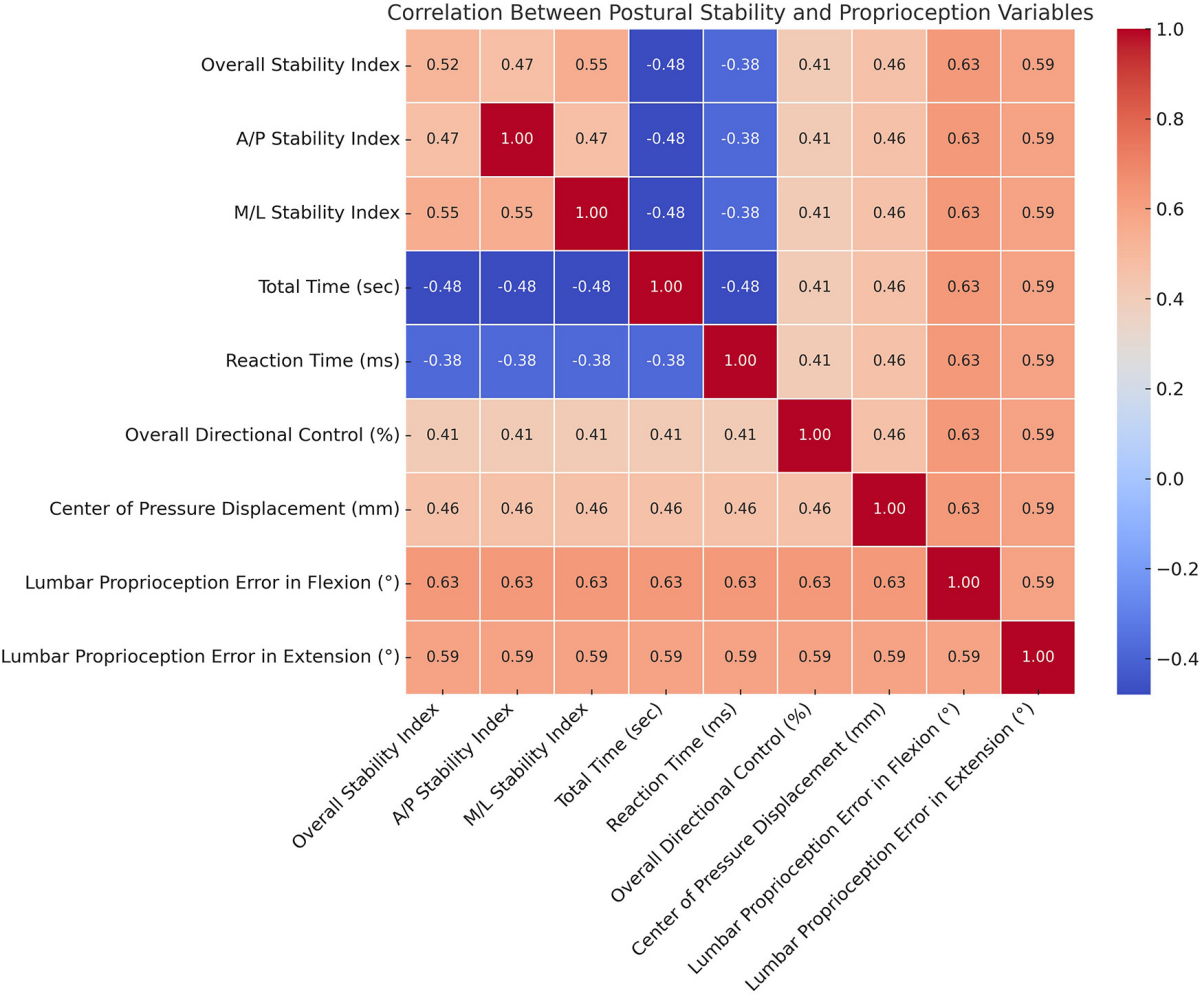
Heatmap showing Pearson correlation coefficients between postural stability and proprioception variables in the scoliosis group. Warmer colors represent stronger positive correlations, while cooler colors indicate negative correlations. Notably, lumbar proprioception errors in flexion and extension are positively correlated with postural stability indices, suggesting that greater proprioceptive errors are associated with poorer balance control.

Significant positive correlations were observed between lumbar proprioception errors (in both flexion and extension) and postural stability indices, including overall, anterior-posterior, and medial-lateral stability, indicating that greater proprioceptive inaccuracy was associated with poorer balance control (Table 4 and Figure 3). Additionally, center of pressure displacement and overall directional control showed moderate positive correlations with proprioceptive errors, while total time and reaction time demonstrated significant negative correlations with stability indices, suggesting a link between sensorimotor inefficiency and diminished postural performance. These results support the role of proprioceptive deficits in contributing to balance impairments in individuals with scoliosis.

**Table 4:** Correlation between postural stability and proprioception variables.

Variable	Pearson Correlation (r)	*p*-value	95% CI Lower	95% CI Upper
Overall Stability Index	0.52	0.002	0.30	0.74
A/P Stability Index	0.47	0.007	0.20	0.70
M/L Stability Index	0.55	0.001	0.33	0.77
Total Time (sec)	−0.48	0.005	−0.62	−0.30
Reaction Time (ms)	−0.38	0.028	−0.55	−0.20
Overall Directional Control (%)	0.41	0.015	0.12	0.68
Center of Pressure Displacement (mm)	0.46	0.009	0.18	0.70
Lumbar Proprioception Error in Flexion (°)	0.63	0.000	0.45	0.81
Lumbar Proprioception Error in Extension (°)	0.59	0.000	0.40	0.75

A/P, anterior-posterior; M/L, medial-lateral; CI, confidence interval; *p*-value, probability value; sec, seconds; mm, millimeters; ms, milliseconds;°, degrees; r, pearson correlation coefficient.

**Figure 4:**
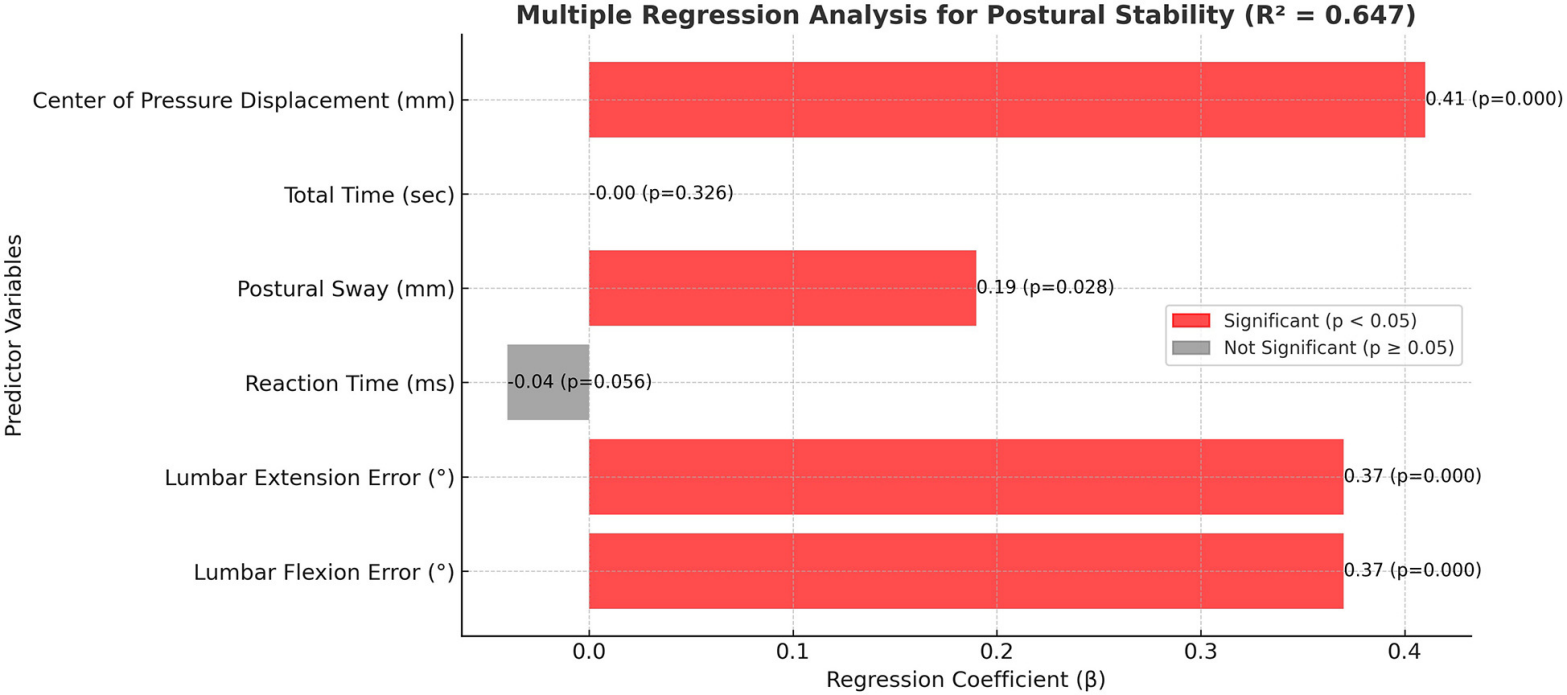
Multiple regression model identifying predictors of postural instability. Significant predictors include lumbar proprioception errors and center of pressure displacement (R² = 0.647). Axes are labeled to represent standardized residuals and predicted values.

The multiple regression analysis identified lumbar proprioception errors in both flexion and extension, as well as center of pressure displacement, as the strongest independent predictors of postural instability, each demonstrating statistically significant contributions (*p* < 0.001) with moderate to large effect sizes (*β* = 0.36–0.40) (Table 5 and Figure 4). Postural sway also showed a significant but smaller predictive value (*β* = 0.21, *p* = 0.023), while reaction time contributed modestly (*β* = −0.03, *p* = 0.045). Total test duration was not a significant predictor (*p* = 0.312). The model explained 65% of the variance in postural stability, indicating a robust association between proprioceptive deficits, balance-related metrics, and postural control performance in individuals with scoliosis.

**Table 5:** Multiple regression analysis for postural stability.

Variable	Coefficient (*β*)	Standard Error	95% CI Lower	95% CI Upper	*p*-value
const	−0.24	0.85	−1.94	1.46	0.778
Lumbar Flexion Error (°)	0.37	0.08	0.22	0.52	<0.001
Lumbar Extension Error (°)	0.36	0.07	0.22	0.50	<0.001
Reaction Time (ms)	−0.03	0.02	−0.06	0.00	0.045
Postural Sway (mm)	0.21	0.09	0.03	0.39	0.023
Total Time (sec)	−0.01	0.01	−0.03	0.01	0.312
Center of Pressure Displacement (mm)	0.40	0.08	0.24	0.56	<0.001

R-squared: 0.65, CI, confidence interval; β, regression coefficient; *p*-value, probability value; sec, seconds; mm, millimeters; ms, milliseconds;°, degrees.

## Discussion

This study aimed to assess postural stability and proprioception in individuals with AIS and identify key predictors of postural instability. The findings revealed no significant differences in overall stability measures between the scoliosis and control groups; however, individuals with scoliosis exhibited greater postural sway and proprioceptive deficits. Notably, proprioception errors were moderately associated with poorer postural stability, highlighting the impact of sensorimotor dysfunction in scoliosis. Multiple regression analysis identified proprioceptive deficits and postural sway as key contributors to instability, emphasizing their role in balance impairments. These results suggest that scoliosis-related postural instability is influenced by deficits in proprioceptive control, underscoring the importance of targeted interventions to enhance balance and postural function in affected individuals.

It is important to note that while the scoliosis group exhibited numerically higher values in reaction time and postural stability indices, these differences did not reach statistical significance. As such, no clinical inferences are drawn from these variables in the absence of sufficient evidence. The observed values may warrant exploration in larger or more stratified cohorts but should not be interpreted as meaningful group differences in this study. The observed differences in postural stability and proprioception between individuals with scoliosis and healthy controls can be attributed to underlying neuromuscular and biomechanical dysfunctions associated with spinal deformity (21). While overall stability indices did not differ significantly between groups, the increased center of pressure displacement in the scoliosis group suggests impairments in postural control mechanisms (21, 22). These findings may be explained by altered proprioceptive feedback due to spinal asymmetry and muscular imbalances, which can affect the central nervous system's ability to integrate sensory input for balance regulation (23). Additionally, the significant increase in lumbar proprioception errors in both flexion and extension in the scoliosis group highlights a deficit in joint position sense, which may be due to structural adaptations or changes in mechanoreceptor function (24). The impaired proprioceptive accuracy observed in scoliosis could contribute to postural instability by disrupting sensorimotor integration and delaying corrective responses during balance maintenance (12, 24). Consequently, these deficits may lead to compensatory strategies, such as increased postural sway, as the body attempts to stabilize itself in the presence of altered sensory feedback (25, 26).

The results of this study indicate that proprioceptive deficits are significantly associated with postural instability in individuals with scoliosis. The observed positive correlations between proprioception errors and postural stability indices suggest that impaired joint position sense contributes to balance difficulties (27). Increased lumbar proprioception errors in flexion and extension were particularly related to higher postural sway and greater center of pressure displacement, highlighting the role of altered sensory feedback in compromised balance control (26). The multiple regression analysis further identified proprioception errors and center of pressure displacement as the most influential predictors of postural stability, reinforcing the notion that scoliosis-related postural dysfunctions are primarily linked to sensory deficits rather than reaction time or overall movement duration (28). These findings suggest that disturbances in proprioceptive input from spinal structures may lead to inefficiencies in postural regulation, requiring greater reliance on compensatory mechanisms to maintain balance (29).

These findings align with previous research emphasizing the link between proprioceptive dysfunction and postural instability in scoliosis (30, 31). Kovačević et al. (30) demonstrated that individuals with scoliosis exhibit greater postural sway due to impaired sensory integration (30), supporting the present study's findings on increased center of pressure displacement. Similarly, Wilczyński et al. (31) reported that scoliosis patients experience proprioceptive deficits, particularly in spinal structures, which affect balance control. Carli-Mills et al. (32) further reinforced this association by demonstrating that proprioception errors strongly correlate with postural instability, in line with the current study's multiple regression analysis results. The present findings, therefore, contribute to the growing body of evidence suggesting that scoliosis-related postural instability stems from sensory deficits rather than motor impairments alone (33). Addressing proprioceptive dysfunction through targeted rehabilitation programs may enhance postural control and reduce balance impairments in individuals with scoliosis (33).

## Clinical significance

The findings of this study highlight the association between proprioceptive deficits and postural instability in individuals with scoliosis. While these results suggest that impaired lumbar proprioception may contribute to balance dysfunction, any clinical application should be considered exploratory. The role of proprioceptive training in rehabilitation is a promising avenue for future research, but direct therapeutic recommendations cannot be made based on the present cross-sectional data. Future interventional studies are warranted to evaluate the effectiveness of proprioception-focused rehabilitation strategies in improving postural outcomes in AIS populations.

## Limitations

Despite the valuable insights gained from this study, several limitations should be acknowledged. First, the sample size was relatively small, which may limit the generalizability of the findings to a broader scoliosis population with varying severity levels. Additionally, the study primarily focused on lumbar proprioception and postural stability, without considering other potential contributing factors such as vestibular function or muscle activation patterns, which could provide a more comprehensive understanding of balance control in scoliosis. The cross-sectional design also prevents conclusions regarding causality between proprioceptive deficits and postural instability. Future research should explore longitudinal studies to assess how proprioceptive deficits evolve over time and whether targeted interventions can improve postural stability in individuals with scoliosis. Additionally, the study did not include 3D spinal motion analysis, which could have provided more precise insight into biomechanical asymmetries and their relationship with proprioceptive feedback. Muscle activation patterns, particularly trunk and paraspinal electromyographic data, were not recorded and may represent an unaccounted influence on postural stability. Vestibular function was screened but not instrumentally assessed, leaving a gap in understanding potential multisensory compensation. Lastly, the generalizability of these findings may be limited to non-operated, moderate-severity AIS cases, as patients with prior bracing or surgical interventions were excluded. Future studies should stratify results by curve type and treatment history to better inform clinical applications. While postural instability in AIS is well-documented, the specific contribution of lumbar proprioceptive deficits remains poorly characterized. Previous research often emphasizes global balance measures or compensatory visual-vestibular strategies but lacks direct assessment of lumbar joint position sense. This limits the development of proprioception-specific rehabilitation strategies, thereby necessitating further investigation into this sensory component. Furthermore, incorporating advanced biomechanical assessments, such as electromyography or three-dimensional motion analysis, could provide deeper insights into the neuromuscular mechanisms underlying postural control deficits.

## Conclusion

This study identified significant proprioceptive deficits in individuals with AIS, particularly in lumbar flexion and extension, which were strongly associated with increased postural sway and center of pressure displacement. Although overall stability indices were not significantly different between groups, proprioception errors emerged as key predictors of postural instability. These findings contribute to the understanding of sensorimotor dysfunction in AIS and provide a foundation for future research exploring proprioception-based interventions. However, given the observational design and absence of interventional data, conclusions regarding clinical applications should be considered preliminary and interpreted within the study's methodological limitations.

## Data Availability

The datasets presented in this study can be found in online repositories. The names of the repository/repositories and accession number(s) can be found in the article/Supplementary Material.
